# Friendship, hope and communication during second-wave and postfeminism: Gillian E. Hanscombeʼs *Between Friends *and Sally Rooneyʼs *Conversations with Friends*

**DOI:** 10.12688/openreseurope.20794.1

**Published:** 2025-08-07

**Authors:** Andrea Anđelinić

**Affiliations:** 1 Faculty of Humanities and Social Sciences, University of Zagreb, Zagreb, 10000, Croatia

**Keywords:** second-wave radical feminism, postfeminism, neoliberalism, epistolarity, communication, hope

## Abstract

Hanscombe's 1982 epistolary novel *Between Friends* offers insight into the main ideas, conflicts and blind spots of Anglo-American second-wave (radical) feminism. Highly contested by its successors, the second wave of feminism in the Anglo-American context fiercely debated issues, such as sex and class, female separatism and political lesbianism. These are the matters *Between Friends* addresses via a heated discussion between four women on matters such as misogyny, autohomophobia, motherhood and queer love. The answers they advocate for, such as forming a utopian lesbian enclave, abandoning all male children, and aborting male feti, show the tendency of second-wave feminists to seek grand, albeit reductive, solutions to the issues posed by the patriarchal capitalist system. The protagonists of Sally Rooney's 2017 novel *Conversations with Friends* seem to be just as painfully aware of their own class and gender hindrances as their second-wave sisters, but the tone of their conversations is skeptical of 20 ^th-^century metanarratives and refrains from offering any sort of solution to their social positioning. The discontinuity and inconsistency of (digital) communication between the characters in the novel reflects the uncertainty and aimlessness characterising their pursuit of love, social success and economic stability. This research aims to compare these two approaches to friendship and feminism by interpreting both the ideas the characters represent and their modes of communication as a consequence of the shifts in feminist theories caused by late capitalism and its effects.

## Introduction

Gillian E. Hanscombe's 1982 epistolary novel *Between Friends* ^1^ centers on four women – Meg, Frances, Jane and Amy – engaged in lively, albeit written conversation about their day-to-day life and relationships, as well as a livelier debate about feminism, which is the main topic of all of their letters. The reader witnesses their relationships forming, evolving and dissolving during the course of the novel, mostly because of their different relations to feminism and the consequences those relations have on their life choices. The four main characters could be said to represent four different approaches to the topic of feminism in their everyday lives: Meg is a lesbian feminist pondering radical ideas, but unwilling to give up her son in order to succumb to them; Frances is an educated and successful antifeminist in a relationship with a misogynist, Jane is a radical feminist advocating for separatism and living in a lesbian commune and Amy is a heterosexual ally practising progressive feminist ideas in her day-to-day life with her partner Tim. These four women represent quite literally different approaches to a topic as dividing as feminism at a time when these differences hold the potential to cause heated, opinion, and life-changing discussions among feminists. The main character of Sally Rooney's 2017 novel *Conversations with Friends* ^2^, Frances, on the other hand, is a well-educated but economically underprivileged young poet living in Dublin. She and her three friends – Bobbi, Nick and Melissa – represent a different kind of community. Not as tightly knit as Hanscombe's, these characters' conversations rarely develop into discussions, rarely center on social subjects, and are characterised by intermittent, ironic communication styles. Frances herself can be viewed as a postfeminist ^3– 5^ subject whose relationship and communication styles reflect her socioeconomic position as well as the dominant structure of feelings ^6^ that emerged during the development of late-stage capitalism in contemporary Western society.

These two types of characters and communities relate to their historical and political contexts, both emerging at a time of specific social climate: Hanscombe's can be observed as a development strongly related to the politics of the feminism of the second wave and Rooney's as a narrative related to the complex sociopolitical context of the 21 ^st^ century. The formal analysis of these narratives shows exactly how these ideological circumstances affect the narrative outcomes of these novels: the epistolarity of Hanscombe's *Between Friends* relates to the hopeful, engaged second-wave subject communicating and discussing in a devoted, continuous manner, while Rooney's *Conversations with Friends*' usage of short, discontinued forms such as e-mails and texts embody the alienated, cynical subject of postfeminism. Despite these contextual and textual changes, the endings of both novels seem to favour hope and community over the individualistic superiority of irony. These novels show, consequently, that hope, love and friendship emerge in different political climates, are performed and valued differently, but they nonetheless represent integral parts of one's identity and pursuit of a ‘better' future, whatever it may entail for each of them as individuals and as parts of communities. 

## Is friendship (just) personal? Theorising feminism and community in Gillian E. Hanscombe's *Between Friends*

The correspondence of the four characters of Hanscombe's *Between Friends* might offer insight into second-wave feminism, a period of feminist thought and action ranging from the late 1960s to the late 1980s, gaining its now familiar shape primarily in the US and the UK. It is important to note that feminisms of the second wave advocate for diverse ideas, but it is, however, as Judith Evans argues in her 1995 study *Feminist Theory Today: An Introduction To Second-wave Feminism*, agreed upon that the broadest distinction in the second wave of the feminist movement is that between liberal and radical feminism ^1^. Evans defines these antithetical positions but also traces the disparity between what we group as radical feminist voices and their policies. Nevertheless, she manages to outline the main arguments of these approaches, focusing mostly on the plurality of ideas we today label as ‘radical'. Liberal feminism, on one hand, rejects sexual differentiation and embraces similarity as an argument for equality. It aims to reform the existing social system in order for it to be permeable for women, believing that justice is possible under existing social conditions ( 7 on page 29–33). Radical feminisms in the second wave rejected this notion. ^2^

Although radical feminisms of the second wave differ in terms of understanding womanhood, social inequality, means and goals of social change, they agree that radical social change – going “to the root of things“ ( 8 on page 2) – is necessary in order to ever build a just society. Ideas that emerged during this period are deemed simplistic and even laughable – such as lesbian separatism, political lesbianism and the abandonment of all men, including male children, and serve as an indicator of how second-wave feminism believed in the possibility of revolutionary change, even if it did not always know what that change would look like. This belief caused them also to neglect differences – such as racial or class backgrounds that rendered the question of liberation much more complex and non-reducible to dissolving only patriarchal structures that oppress women – but, on the other hand, their insistent trust in unity made possible the type of productive enthusiasm the feminist movement has not been able to reproduce since. This is the social climate contextualizing Gillian E. Hanscombe's novel *Between Friends* which, although published in 1982, serves as a sort of re-examination of the earlier decade – the seventies – marked in the West by growing social justice movements and their success in acquiring space in the public discourse. Feminism and the Gay Liberation Movement are some of the most prominent of the movements that emerged in the 1960s Europe and the US ( 9 on page 1), and feminism especially emerged “out of the anti-imperialist New Left as a radical challenge to the pervasive androcentrism of state-led capitalist societies in the postwar era” ( 11 on page 209). The enthusiasm and hope characterising these decades were thoroughly defeated during the return to conservatism in the 1980s, but the fictional world of *Between Friends* keeps the utopian sentiment of second-wave feminism and the Feminist Liberation Movements alive, as we will show, referencing it both textually and contextually.

As we have stated, three characters in the novel – Meg, Amy and Jane – embody different branches of radical feminism in the second wave. Frances' view, however, differs greatly from her friends' and her personal lifestyle and life decisions allow her to reject feminism altogether. Claiming that she is liberated as it is, living in an informal union with a man she is not economically dependent on and characterises him as progressive and liberal, Frances is convinced that feminism has nothing left to do for her. Her partner Jim is the one who did not want marriage, insisted on her financial independence, and did not want any children – he is the picture of a contemporary man. She is the woman liberal feminism teaches us we can become – she is proof, to herself at least, that by the 1980s, feminism has fulfilled most of its goals. She celebrates feminist accomplishments such as the right to vote, the right to an abortion and pay equality, enjoys the fruit of other women’s labor, but refuses to entertain the possibility that gendered social injustice is still a problem in most women’s lives. “I must have been right all the time” – she says in a letter to Meg ( 1 on page 14) – “in spite of all your intellectualisations – feminism is just another name for lesbianism, when it comes down to it. I support women's issues, as you know – abortion, equal pay, all the rest of it – but I don't support the rejection of men as human beings, which it seems to me is what lesbians do“ ( 1 on page 14).

Frances is an emancipated, independent woman who refuses to see any link between the personal and political – “I want our friendship to be personal – not overlaid and twisted about and distorted and intellectually manipulated by a creed that is not my own” ( 1 on page 8), she tells Meg. Frances' refusal to admit Meg's sexuality bothered her as well as her refusal of Meg's friendship after Meg was raped by Frances' partner show how personal and political are at odds in Frances' mind. Instead of standing in solidarity with her sexually violated closest friend, Frances chooses to isolate and succumb to her fear of lesbianism, staying with her partner, and refusing any letters or proof of her husband’s infidelity. Therefore, her personal fears prompted her to make what is in fact a political decision: to stay quiet about sexual assault and continue supporting the man responsible for it. The liberal feminist ideal of the emancipated woman is revealed in *Between Friends* as plagued exclusively with self-interest and unwilling to offer solidarity with others, dependent entirely on the comfort liberal society secures for one playing by its rules.

On the other hand, Meg, Jane and Amy form their own perspectives on the feminist ideas of the second wave. While Jane advocated for separatism, Amy was more of a reformist, believing in the ability of feminism to reshape the structures of power as well as those reinforcing them. Meg functions as a narrative bridge between these two conceptions of radical feminism, always asking questions and eager to forge a middle path, but never questioning the necessity of extreme societal or political change in building a more just reality.

Friends exchange lengthy letters documenting not only their different stances on social justice but also their fervour and enthusiasm with which they approach social change. Theory and practice are shown in *Between Friends* not to be exclusive but to co-exist: to theorise – to write, discuss, speak is, for these characters, a revolutionary practice. Their daily lives reflect this practice: Meg lives in a homosexual partnership and has a child conceived via IVF, a method in which she has become an expert and uses this expertise to fertilise other women (mostly lesbians). Amy and her husband Tim practice sex without penetration, and both believe that penetration is a practice that enforces male dominance over women. Tim is a reformed sexist, part of a male anti-sexist group unlearning male behaviours that ensure their unjust treatment of women. Jane is a divorced political lesbian living in a self-sufficient lesbian community whose activity focuses on various modes of organising to educate and politicise women. They all believe in the revolution, talk about it, write of it, and dedicate their lives to it, although with different intensities. Their personal lives reflect their creative, open-ended feminist theories: they exclude men (except for those willing to change and better themselves); they contribute to the improvement of the lives of women, seeking not only to educate others, but to educate themselves, always posing and answering questions, and always intellectually and emotionally curious. They come up with grand ideas and plans to execute them: they move in a house to live together, forming their own little community that fosters the ideals of second-wave radical feminism. They plan and organise a big protest against marriage with thousands of women – and some men – burning their marriage certificates and/or divorce papers, as they all believe the end of marriage will end in a certain kind of oppression. They converse, but they also act, choosing to strongly believe in the power of feminism to change the world, to bring about ‘the revolution'. This utopian sentiment characterises the radical feminism of the second wave and it ideologically correlates with modernism, such as Jameson ^12^ describes it, and its belief in progress as one of the big metanarratives of our world.

Within this fictional universe it is possible as well as necessary to envision a better version of reality, and this reality is shaped within the context of a community – a concept that signifies not only a group of people bound by “a sense of togetherness and shared values” ( 13 on page VII), but also by a proclivity to ‘think’, or theorise the very concept and the values they are thought to share ( 14 on page 98, 13 on page XII). This is exactly the communal action characterising the friendly correspondence of *Between Friends*: they theorise their own existence within the structures of society, shaping their values in the symbolic communal space of letter writing where communication and discussion are welcome and fruitful, effectively making them a community. This is also a symbolic space that emphasises empathy and care as crucial factors of feminist politics: when Meg is raped, her friends not only write letters to show their compassion and offer comfort, but they also reach out to Frances, the woman whose husband raped Meg, to offer their support. They insist on writing to her even when her replies make her denial and complete disconnection from the topic of her partner’s guilt obvious. Jane even goes so far to seduce Jim, photograph it and send it to Frances, an act she interprets as “uncivilised” and “destructive” ( 1 on page 170), failing to recognise this as an attempt of radical solidarity on Jane’s part. “Uncivilised” and “destructive” are for Jane necessities in building a better world by her standards, so the criteria of the heteronormative, liberal society advocating for civility and constructive reform aren’t what she adheres to. These standards are not what a radical feminist community makes, so by rejection of civility and by embracing destruction as a guiding principle which makes revolutionary change possible, Jane not only theorises her ‘community’, but shows how its principles work in practice to actively reject the values it rebels against. She is “destructive”, but as a means of helping her friends, as a means of proving what she deems just. As a community theorist, Jane is exclusive; her idea of a community excludes men of all ages and backgrounds. To form a community, then, for Jane, means not only to include (those who belong) but also to exclude those who do not, and not even by their choices or values, but by their gender alone. This criterion deems theorising further impossible if done by men: it is deterministic in the same way misogyny is, but having its logic reversed and brought to an extreme might, for the reader (be it the inside (letter) reader or the external (novel) reader), enabling unmasking its weaknesses. Meg and Amy, on the other hand, adhere to standards of “civility” and reject Jane’s “destructive” principles, theorising their community as a symbolic space that cultivates growth and change, even within men. They view themselves, as well as men, as “processual” beings ( 15 on page 56), who are always able to develop and revise their previous versions. By placing hope in the process of human development, both theoretically (by writing) as well as practically (by doing and by writing-as-doing), Amy and Meg serve to prove that the subject of the second-wave feminism is not just a laughable separatist, but a (self-)conscious individual willing to act as well as theorise, creating a symbolic (and also literal) space where ‘community’ is – just like an individual themselves – an open-ended, continuously forming and evolving organism. This processual idea of community proves victorious at the end of the novel, when characters move into a house together with their community members, and even Jane, after a lengthy discussion, evolves to a less “destructive” version of herself, one which concedes to love and relinquishes her deterministic principles by joining a community that includes men: “yes to you, yes to Simon, yes to moving to London, yes to life” ( 1 on page 175), she concludes, choosing, at last, to live. Because to live, in this community, is to evolve.

## “More comfortable with critique than endorsement”: the making of a fictional postfeminist subject in Sally Rooney's *Conversations with Friends*

The shift between modernism and postmodernism, between the insistence of a single metanarrative and the awareness of their multiplicity, precedes and explains to a degree changes in feminist theories. Radical feminism of the second wave has not yet abandoned metanarratives, unlike so-called postfeminism. As Barbara Creed explains:

Whereas feminism would attempt to explain that crisis [of legitimation that Lyotard has described] in terms of the workings of patriarchal ideology and the oppression of women and other minority groups, postmodernism looks to other possible causes – particularly the West’s reliance on ideologies which posit universal truths – Humanism, History, Religion, Progress, etc. While feminism would argue that the common ideological position of all these ‘truths’ is that they are patriarchal, postmodern theory would be reluctant to isolate a single major determining factor. ( 16 on page 52)

Rosalind Gill, a theorist of postfeminism, a term used to describe the plurality and oftentimes contradictory views on feminism in contemporary Western society, qualifies it not as a movement or a theory, but as a sensibility adjacent to neoliberalism, which dictates it ^4^. According to Gill, the principles of neoliberalism, most of all the fetishisation of individuality and individual responsibility, have impacted mainstream feminism, giving it the shape it has today ^4, 5^. This results in passiveness that characterises our political climate: hope is scarce in a world fixated on personal responsibility and psychology, and with all grand narratives abandoned, with big social movements and the social charge they brought about fading, the utopian world of second-wave feminism seems to be not only distant and impossible, but laughable. This is the climate – sensibility – characterising Sally Rooney's 2017 novel *Conversations with Friends*. This is also a connection discussed thoroughly in José Carregal-Romero's 2023 article titled “Unspeakable Injuries and Neoliberal Subjectivities in Sally Rooney’s *Conversations with Friends* and *Normal People*”, which interprets the causes of characters’ behaviours as influenced by neoliberal subjectivity. Rooney’s characters engage in conversations on radical politics and alternative lifestyles, but they nonetheless struggle with emotional intimacy, and their relationships are affected by neoliberal constraints such as class-based distinctions and prejudice, as well as gender and sexual hierarchies often established through (self-)objectification” ( 17 on page 214).

*Conversations with Friends* focalises the main character and narrator, Frances, a young student, a poet, a Marxist, as some characters in the novel claim, working precariously for literary agency during the first part of the novel, after which she is unemployed. Her relationships with her friends – Bobbi, Melissa and Nick – are a far cry from the communal structures built by Hanscombe's characters. Despite being her closest friends, Frances finds no ability to talk to them openly and earnestly about her financial distress, serious health issues she is experiencing or her emotional state. She communicates in a discontinued, occasional manner, enclosing herself in a private world of self-inflicted suffering. From Carregal-Romero's perspective, the neoliberal self is alienated not only from others, but also from itself, and the powerlessness to change those modes of articulating (or not articulating) emotion forces it into silence ( 17 on page 218). Other characters share this characteristic, at least to a degree, also noting that Frances is the one to never talk openly about her emotions. Frances' isolation during her endometriosis outbreak and diagnosis reaches such a degree that she struggles to find any value in her life, causing her to harm herself and close off to others even more drastically. “I was a damaged person who deserved nothing” ( 2 on page 213), she concludes while deliberating death. This accentuated worthlessness and depression should be read as a symptom of Frances' social position: even though she is a successful poet and an educated young woman, her sense of worth stems from what she could not achieve – financial stability, substantial, stable relationships with others, open communication, a sense of community. She, in spite of privileges setting her up for a ‘good', ‘successful' adult life, has been rendered unable to achieve this ‘success' due to her emotional and physical difficulties ^3^ as well as, more importantly, by her precarious lifestyle. She is then, in this way, a product of context, and decisions impacting her relationships with others are greatly influenced by her social position and financial status. Her decision to publish her first short story, one about her friend Bobbi, is mostly influenced by the fact that she is *literally* starved for food. Thus, her career as well as her relationship with a longtime friend and lover have both been made economic rather than personal or interpersonal decisions. Frances' story once again shows exactly what was shown when interpreting her namesake's decisions in Hanscombe's novel – that the personal and the political are not only intertwined, but dependent upon one another. Rooney's Frances, having to risk betraying a valued friendship to survive, shows, in part, how ‘community' has let her down (because ‘community', for Frances, was never an option to begin with). The thought of borrowing money, let alone of living communally or sharing resources – all ideas Hanscombe's characters argue for and make true in their little ‘commune' at the end of *Between Friends* – has not even crossed her mind. These kinds of practical solidarity are not a part of Rooney's Frances' feminism anymore. They are long gone, replaced by a growing insistence on individuality and the distrust in social change in the age of neoliberalism and postfeminism, both of which insist on laying the blame for lack of success on an individual unable to endure the “competitive“ social relations forced upon them ( 18 on page 948), masking the effects of the “affective“ power of neoliberal ideology over an individual's choices and social position ^19^.

Although the characters in the novel, which some interpret as typically millennial and influenced by the Internet ^17^, never miss a chance to label themselves as ‘this and that', Frances doesn't label herself as feminist, and neither do other characters in the novel. This is not to say that they do not share feminist ideas – they touch upon different feminist topics and Frances does disclose that she is a part of a feminist university group – but the idea of labelling oneself as feminist seems somehow reductive to these characters. It seems as though it is implied, given their opinions and comments, that they are (all?) feminists, but there seems to be no need to proclaim one's feminism, or ponder change, let alone ‘the revolution'.

There is no enthusiasm about social change, no big ideas being thrown around in conversation – the characters touch upon political subjects all the time, but they do so in a detached, almost ironic way, reluctant to adhere to any narrative, embracing only their skeptical postmodernist logic. “More comfortable with critique than endorsement” ( 2 on page 241), is how Frances describes her attitude towards political issues, capturing the dominant sensibility of her generation. She has no hope or grand plans for her future, and she notes: “I had no plans as to my future financial sustainability: I never wanted to earn money for doing anything. (...) I certainly never fantasised about a radiant future where I was paid to perform an economic role” ( 2 on page 23). She is indifferent to money as long as her primary needs are met, and she claims her “disinterest in wealth” is “ideologically healthy” ( 2 on page 23). However, as was already mentioned, when faced with the fact that the performance of an ‘economic role' isn't a choice to be made, but a material obligation to be fulfilled, Frances is forced to dismiss her irony and accept her role in spite of cherished “ideological health”. Thus, Žižek's conclusion that “cynical distance is just one was to blind ourselves to the structural power of ideological fantasy: even if we do not take things seriously, even if we keep an ironical distance, *we are still doing them*” ( 20 on page 30), rings particularly true in our heroine’s case. Her friend Bobbi who, on the other hand, at one point proclaims “depression is a response to late capitalism” ( 2 on page 124), proving to be completely aware of the effects of socioeconomical context on the individual – just as Frances is – but choosing a rather different approach than her. Bobbi is a proactive, vocal participator and leader in university groups (for example, she leaves a feminist university group after they invite a speaker that supports the invasion of Iraq, whereas Frances, even though privately agreeing with Bobbi, remains a part of the group ( 2 on pages 63–64)). This active and oftentimes over-confident position Bobbi takes can in part be a reflection of her upper-class background Frances deemes made her politics seem performative to those of the same class as “other wealthy people recognised her as one of their own” ( 2 on page 95). While Frances proclaims herself “fearful” and “out of place” ( 2 on page 95), she sees Bobbi’s position as an upper-class intellectual with working-class politics as something that can provide her with enough cultural capital to make herself feel welcome in any social context. The proactiveness and ability to imagine a different, better world for herself and others, then, in Frances’ eyes, stems from one’s own socioeconomic background: to imagine wealth, you have to have experienced it, feel comfortable in it, and live it. You cannot have only theorised it, as that would only make you “fall silent” ( 2 on page 96) in the face of injustice.

Therefore, Frances, as well as other characters in the novel – such as Nick – seem to be painfully aware of their own social contexts and the injustices that arise from them, but they show no intent to change their social situations and no set of political beliefs that would compel them to believe in meaningful social change. They seem to be in a state of “reflexive impotence” ( 21 on page 21), unable to act or even imagine significantly different lives for themselves. Frances-the-narrator never delves into the details of their conversations, and all their remarks remain at the surface level, always presented matter-of-factly. Their discussions are mostly concluded with ironic comments, which pose them a safe distance from the matter they are discussing. Gill ^4^ considers this ‘ironic distance' to be one of the characteristics of a postfeminist subject, while Carregal-Romero concludes this mechanism is mostly damaging for Rooney's characters: their irony is exposed in *Conversations with Friends* as a strategy that “equals (self-)deception, and only becomes a symptom of unease and discomfort” ( 17 on page 219). It seems that their conversations and discussions serve not to learn from one another, but to humiliate one another intellectually, and thus socially, as they all co-exist in a social environment that values intellectualism – and individualism – above all. At those times, these mechanisms are proven futile and dangerous for the characters that new possibilities emerge: “change is produced on interpersonal levels, when characters confront their self-abjection, [and] achieve positive human connection” ( 17 on page 215). Our aim is to further interpret the ways in which this human connection is hindered within the novel and the mechanisms used to shape this kind of inability to communicate, attributed to the effects of neoliberalism and postfeminism on these characters. As silence is considered to be Frances’ main communicative strategy, we would also like to explore what narrative models frame Frances’ interpersonal habits when she *is* actively trying to converse and how her communication style changes, as well as compare the communication styles and narrative structures of *Conversations with Friends* with its predecessor, *Between Friends*.

## “You can't just say you're ‘anti'-things”: narrative strategies and their implications in Gillian E. Hanscombe's *Between Friends* and Sally Rooney's *Conversations with Friends*

Rooney's novel, besides classical first-person narration, also incorporates text messages and emails characters sent to one another. These messages range in topics from flirting to philosophical comments, but they are always only brief and passing; often witty one-liners that serve mostly Frances' need to have control over her own image. This connects to the neoliberal and postfeminist imperative of self-surveillance and control Gill ^4, 5^ claims that people have internalised to better fit their role in the world of late capitalism. ^4^ Frances' unwillingness to participate in oral discussions (where she is almost always quiet, but listens closely as Bobbi defends her points, which are, one presumes, shared) and her overeager analysis of text messages between her and Nick and her and Bobbi contributes to the conclusion she is far more preoccupied with maintaining a certain image (that of a smart, educated person, a good writer etc.) than with actually discussing social or philosophical issues. This insistence on self-surveillance and self-image control makes Frances a postfeminist subject *par excellence.* (That being said, all her self-reflexivity serves not to distinguish her from this model and prove her to be a “post-ideological” subject ( 20 on page 30), but to show that her cynicism and irony are only masking the fact this is an “(unconscious) fantasy structuring our social reality“ ( 20 on page 30)). 

This is the difference between Rooney's and Hanscobe's conversations – Rooney uses them as an insight into the characters’ fears and compulsions, whereas Hanscombe uses conversations to form character connections. The letters Hanscombe's characters write to one another – the effort that goes into them, the passion they display towards one another, as well as for the topics they argue – are carefully written, detailed and long; they exhibit continuity, which is in line with the ideas they argue and support. The idea that it is worth explaining oneself, proving your points to others, writing another letter when one remains unanswered, going above and beyond to ‘get through' to someone – this is the idea that even feminism has not been nurturing enough lately. This is the idea we have abandoned in the era of postfeminism, where we are sometimes too alienated from one another and too concerned with self-image to engage in meaningful, potentially fruitful conversations. “We cannot develop communal commitments because we cannot think of ourselves as citizens—as active, involved, caring political beings” ( 22 on page 340). Thus, if we do not consider ourselves citizens – do not consider social change at least partly our responsibility – we lose the need to engage in the conversations Hanscombe’s characters insist on in the first place.

The epistolary form Hanscombe uses in her novel is a traditionally female type of writing – one belonging to the intimate sphere, one often focusing on emotions, one that does not regulate streams of thought but encourages it, for it is exactly this kind of seemingly free-flowing thinking and sharing that is most beneficial to the emergence of new ideas. It is exactly via letters, as Margareta Jolly concludes in her 2008 study, *In Love and Struggle. Letters In Contemporary Feminism* ^23^, feminists exchanged ideas and formed bonds with one another. The exchange of letters between four friends makes this text a space where dialogue and communication become not only a possibility, but also a necessity in order to continue believing in and bringing about social change. If there is space for dialogue, there is space for hope, and hope is something that radical feminism of the second wave is abundant. The epistolary form, internally coherent and fragmented at the same time ( 24 on page 183), is used in *Between Friends* as a means of juxtapositioning perspectives, making it possible to examine each character’s arguments and motives side by side. By using this approachable and clear narrative strategy, Hanscombe made it possible for the letters to serve not only as a fictional plot but also as a kind of introduction to practical feminism, including all of its pitfalls and strong places, the strongest of which is exactly the one examined during this analysis: the potential for making lasting friends we can learn from as well as educate, making those small interpersonal steps in the grand world-changing narrative of second-wave feminism. The four autodiegetic narrative perspectives enable the text to decentralise a single character as well as the plot and to make the core of its interest the *conversation* itself. While Rooney's autodiegetic narrative focuses on a single character, making her a hero – albeit a faulty, haunted one – who is enclosed in the narrow fictional universe of her own making, Hanscombe's decision to make her novel a four-person narrative completely devoted to establishing and exploring the characters/narrators' communication with one another gave way to making friendship and community seem possible among these characters. It provided them – as well as the reader – with the narrative continuity and mutuality necessary for creating hope and interpersonal happiness and giving them a happy ending in their own terms.

Rooney's choice to incorporate only short texts and e-mails into the narrative of *Conversations with Friends* functions as proof of the protagonist’s inability and fear of communication, which can be interpreted as a fear of vulnerability that prevents her from connecting to others. We argue that this narrative discontinuity and inconsistency of communication between characters shows not only a fear of vulnerability but can also be interpreted as a socioeconomically conditioned inevitability. The discontinuity and inconsistency of (digital) communication between Frances and the other characters in the novel reflect the uncertainty and aimlessness characterising the pursuit of love, social success and economic stability in a neoliberal society of the 21 ^st^ century. It is, we mean to say, not only the content of the messages, but also the method of communication she adopts that indicates the kind of relationship Frances has to hope for and to friendship. “Capitalism harnesses love for profit” ( 2 on page 180), Frances states in one of her messages to Bobbi, arguing that human connections have as such become commodified in ways that alienate people from one another. This alienation is not something that can be negotiated within the boundaries of traditional relationships, so instead of discussing it – as Hanscombe's characters most likely would – Frances and Bobbi cut their conversation short after Frances' claim she is “anti-love as such” ( 2 on page 180) and Bobbi's retort she can't just say she is “anti-things” ( 2 on page 180). This shows an unwillingness to discuss and a tendency to proclaim, a trait supporting further alienisation of these characters, even if the impulse to rethink conventional relationships is one they share.

The discontinuity of the written communication in the novel, then, reflects Frances' ambivalent stance on love (or human connection) in the age of late capitalism: she indulges in communication, but cuts it off abruptly, often replying to messages briefly and late; she is insistent on forming bonds with others, but cuts them off when she deems herself in an emotionally or economically vulnerable position. She seeks for herself and her friendships a terrain that would enable a shared vulnerability possible only under conditions that relate as little as possible to predetermined gender roles, class hierarchies and heteronormative practices imposed by mainstream society. This becomes possible, as was already noted, towards the end of the novel, as Frances not only chooses to express herself emotionally, but also becomes more economically independent and takes control of her own poetic voice by publishing her work. In this way, the personal and the political are once again proven to be dependent on one another: in order to form and maintain healthy, fulfilling human connection, Frances needs first to claim some economical and cultural capital: the need to ‘open up' to others becomes not only an accidental effect of ‘self-growth' such as late capitalism would have you believing necessary and only achievable through the means of close personal ‘work'. This self-therapeutic practice and the imperative for personal growth seems to be another instance of the postfeminist and/or neoliberal subject, a part of a discourse that has “made us abandon the great realms of citizenship and politics and cannot provide us with an intelligible way of linking the private self to the public sphere (…), replacing this content with a narcissistic self-concern” ( 25 on page 2). *Conversations* then prove this insistence to be insufficient in making a happier person and a healthier communicator: it is through economic stability and cultural capital (in this case) that Frances can finally begin to claim her self-hood and her betterment. This stability can start giving her ample space to breathe under the conditions of late capitalism, opening up the personal and interpersonal effects of this change. The economic and (inter)personal are, once again, joined together in making possible what is deemed Frances’ “evolution from a self-obsessed to a more open and caring individual” ( 17 on page 217).

By joining these aspects – the economic and (inter)personal – the text shows how the effects of neoliberal sensibility cannot be overcome by treating them as separate phenomena. In the same way, the meanings interwoven in the text cannot be treated simply as a product of political and/or cultural context but as effects of textuality. It is not only what Frances says or does not say, but *how* she says and *when* she says it that should interest us. It is, then, symptomatic to dwell on the ending of the novel, the moment Frances’ “evolution” is in ‘full effect’. The novel ends with Frances and Nick, the character whom she has a sexual and friendly relationship with throughout the novel (a relationship that renders visible most of her insecurities regarding her economic position and body image), talking on the phone. The tone of their conversation is optimistic and full of possibility – a far cry from their texts or e-mails throughout the novel. Nick’s economic (he comes from an upper-class family) and cultural (he is an actor married to Melissa, who is a very successful author) capital finally ceases to render Frances worthless in her own eyes, as she has moved from a hopeless, ironic perspective on money, labour and creation to a still precarious, but changing, encouraging position of a published and praised author. In this new position, which helps make care and openness possible, her communication style has changed. The textual choice to have the final chapter of the novel narrated through a phone call, and not a message, then serves as a signal of this ambivalence between the simultaneous desire for human contact and alienation being resolved in favor of communicating. ^5^ The final words of the novel: “Come and get me, I said” ( 2 on page 321) emphasise this choice: Frances, as well as the text itself, chooses to *converse* and participate rather to alienate, for the implications of the latter are far bleaker: they leave one at mercy of the neoliberal Self and its surveillance. 

## Conclusion

Hope and friendship go hand in hand for Hanscombe's characters who serve as literary proof that personal is political – their personal friendships shape their political beliefs, forming them into a community fueled by hope for a better world for all women. This utopian sentiment fell flat in 2017 and today, but friendship is still a sort of kinship with hope: it is exactly through friendship and other kinds of close interpersonal relationships that the protagonist of the novel reveals the value of life. Often read as a Bildungsroman exactly for this reason, *Conversations with Friends,* just as *Between Friends*, finds hope in the community. Both novels are focused on reinterpreting traditional communities and relationships: Hanscombe's through a utopian, somewhat separatist lens of starting new communities unburdened with patriarchal relationships that define our society, and Rooney's through a rethinking of monogamy and conventional boundaries between friendship and romance, such as it is defined in our society. ^6^ Both novels question what it means to communicate, converse, what it means to be vulnerable among those whose love and respect we seek, and what it means to be political in different historical contexts. The at times overbearing intensity of *Between Friends* is juxtaposed to the cold irony of *Conversations with Friends*, the former’s wordy letter correspondence to the latter’s brief and discontinued messages, signaling a shift in the way we communicate, but not in what drives us to converse and to connect to each other. The necessity of human contact seems to incite both of these modes of communication despite the way values, social positions, and the historical moment have changed.

## Ethics and consent

Ethical approval and consent were not required.

## Data Availability

No data associated with this article.
