# Supplementary material for: Friendship, hope and communication during second-wave and postfeminism: Gillian E. Hanscombeʼs Between Friends and Sally Rooneyʼs Conversations with Friends
Source: Open Res Eur. 2025 Aug 7;5:228. [Version 1] doi: 10.12688/openreseurope.20794.1 (PMC12489458; doi:10.12688/openreseurope.20794.1)
Supplement: Peer review history

# Reviewer report: Reviewer response for version 1

**DOI:** 10.21956/openreseurope.22498.r60385

**Published:** 2025-10-07

**Authors:** Zahid Ali Jatoi, Muzamil Syre

**Affiliations:** 1 ELT, University of Southampton Department of Modern Languages (Ringgold ID: 222230), Southampton, England, UK; 2 Sukkur IBA University, Sukkur, Sindh, Pakistan; 3 English, University of Sindh (Ringgold ID: 66702), Jamshoro, Sindh, Pakistan; 4 University of Surrey Faculty of Arts and Social Sciences (Ringgold ID: 105650), Guildford, England, UK

Andrea Andelinic’s article is well-organized and well-toned. Analytically robust in its cross-textual argumentation, its use of epistolary and digital communication shows a convincing design, overemphasized though. It aptly references debates of second-wave feminism and contrasts them with postfeminist skepticism. It examines the contradictions and blind spots in either wave of feminism. It allows the tensions between eras and ideologies to remain unresolved and critiques both the texts without dismissing them.

If any, are all the source data and materials underlying the results available?

Yes

Is the work original in terms of material and argument?

Yes

Is the work clearly and cogently presented?

Yes

Is the argument persuasive and supported by evidence?

Yes

Does the research article contribute to the cultural, historical, social understanding of the field?

Yes

Does it sufficiently engage with relevant methodologies and secondary literature on the topic?

Yes

Reviewer Expertise:

English Literature ( Muzamil Syre) and English Language Education ( Dr Zahid Ali Jatoi)

We confirm that we have read this submission and believe that we have an appropriate level of expertise to confirm that it is of an acceptable scientific standard.

# Reviewer report: Reviewer response for version 1

**DOI:** 10.21956/openreseurope.22498.r60379

**Published:** 2025-10-01

**Authors:** Vishnu Kumar Sharma

**Affiliations:** 1 JECRC University, Jaipur, Rajasthan, India

Title of the Article:

*Friendship, hope and communication during second-wave and postfeminism: Gillian E. Hanscombeʼs Between Friends and Sally Rooneyʼs Conversations with Friends*

 Summary of the Article:

 This article offers a comparative reading of Gillian E. Hanscombe’s Between Friends (1982) and Sally Rooney’s Conversations with Friends (2017), situating them within the ideological shifts from second-wave radical feminism to postfeminism. The study foregrounds friendship and communication as key categories for understanding feminist subjectivity across two very different socio-historical contexts: the utopian and often radical hopes of second-wave feminism and the fragmented, ironic, and neoliberal sensibility of postfeminism.

 By analyzing Hanscombe’s use of the epistolary form as a space of continuity, dialogue, and communal hope, the article contrasts it with Rooney’s fragmented digital exchanges (emails and texts) that reflect postfeminist alienation, reflexive irony, and neoliberal individualism. Importantly, the author highlights how both novels, despite different contexts, end by affirming hope and community, albeit in different registers. The study combines literary analysis with feminist theoretical frameworks (radical vs. liberal feminism, postfeminist sensibility, neoliberal subjectivity) to show how communication strategies mirror ideological positions.

 Evaluation Against Criteria:

**Originality of material and argument – Yes**

 The article makes an original contribution by juxtaposing Hanscombe’s relatively under-discussed Between Friends with Rooney’s Conversations with Friends, thereby illuminating continuities and ruptures between feminist literary imaginaries. The comparison is innovative in scope and draws together feminist theory, cultural analysis, and narrative strategies.

**Engagement with methodologies and secondary literature – Yes**

 The author demonstrates a strong command of relevant feminist theories (Evans, Gill, Fraser, Creed, Žižek, etc.) and situates the novels within critical debates about feminism, postfeminism, and neoliberalism. The article integrates theoretical sources into the textual analysis smoothly and convincingly.

**Clarity and cogency of presentation – Yes**

 The article is well-structured, logically organized, and clearly written. Complex theoretical points are presented accessibly, with careful transitions between literary evidence and conceptual framing.

**Persuasiveness and evidence – Yes**

 The argument is persuasive and supported with detailed textual analysis. Quotations from both novels are aptly chosen, and theoretical claims are substantiated with secondary scholarship. The parallels drawn between narrative form (letters vs. digital messages) and ideological stance are particularly convincing.

**Availability of data/materials – Yes**

 As a work of literary and theoretical analysis, the article relies on published novels and secondary sources, all of which are cited appropriately. There is no issue with data availability.

**Contribution to cultural, historical, and social understanding – Yes**

 The article makes a valuable contribution to feminist literary studies and cultural criticism. It offers insights into how forms of communication reflect broader socio-political contexts and evolving feminist sensibilities. By connecting literary form with ideology, it enhances our understanding of both historical feminist movements and contemporary postfeminist conditions.

**Strengths**

 Innovative comparative framework that bridges second-wave and postfeminist contexts.

 Excellent integration of literary form (epistolarity, digital communication) with feminist theory.

 Balanced treatment of the novels, avoiding over-romanticization of second-wave utopianism while critically analyzing postfeminist irony.

 Clear writing style that is accessible to both literary scholars and feminist theorists.

 Timely and relevant in light of ongoing debates about feminism, neoliberalism, and literary form.

**Suggestions for Improvement (Minor)**

 The article could benefit from a slightly expanded reflection on intersectionality (race, class beyond Frances’ working-class precarity, sexuality in Rooney’s text) to further nuance the feminist contexts.

 While the conclusion successfully links both novels through hope and community, a sharper articulation of what this comparison contributes methodologically to feminist literary criticism as a field would strengthen the broader scholarly impact.

**Recommendation**

 This article is scientifically sound and ready for acceptance. It presents an original, well-supported, and cogently argued analysis that will be of interest to scholars of feminist literary studies, cultural criticism, and comparative literature. Only minor elaborations could enhance its scope, but no major revisions are necessary.

 Decision: Accept.

If any, are all the source data and materials underlying the results available?

Yes

Is the work original in terms of material and argument?

Yes

Is the work clearly and cogently presented?

Yes

Is the argument persuasive and supported by evidence?

Yes

Does the research article contribute to the cultural, historical, social understanding of the field?

Yes

Does it sufficiently engage with relevant methodologies and secondary literature on the topic?

Yes

Reviewer Expertise:

Linguistics, Typographic-semantics, Feminism, Diaspora Writings

I confirm that I have read this submission and believe that I have an appropriate level of expertise to confirm that it is of an acceptable scientific standard.

# Reviewer report: Reviewer response for version 1

**DOI:** 10.21956/openreseurope.22498.r57849

**Published:** 2025-08-27

**Authors:** Madugula Raju, Mrunalini Sasanka

**Affiliations:** 1 B V Raju Institute of Technology, Medak, Telangana, India; 2 ENGLISH, NARSAPUR, MEDAK, B V Raju Institute of Technology, TELANGANA, India

This article presents a well-structured methodology, with its thematic ideology and concept carefully developed. All the manifested characters in it have been explicitly portrayed. Reminiscent feelings among characters are developed and designated in the article that are quite necessary for the modern world. The scientific reasons and logical balances between characters depicted in the article are well versed to make a psychological relationship and scientific approach would surely navigate. 

 I would indeed recommend it for the next level.

If any, are all the source data and materials underlying the results available?

Yes

Is the work original in terms of material and argument?

Yes

Is the work clearly and cogently presented?

Yes

Is the argument persuasive and supported by evidence?

Yes

Does the research article contribute to the cultural, historical, social understanding of the field?

Yes

Does it sufficiently engage with relevant methodologies and secondary literature on the topic?

Yes

Reviewer Expertise:

Critical and analytical dimensions of the neurolingutics and cognitive patterens of thought and mind. I am also certified Internaltional Critical Thinker published many research article over copious databases.

We confirm that we have read this submission and believe that we have an appropriate level of expertise to confirm that it is of an acceptable scientific standard.
